# Contribution of Python-based BERT software for landslide monitoring using Electrical Resistivity Tomography datasets. A case study in Tghat-Fez (Morocco)

**DOI:** 10.1016/j.dib.2022.108763

**Published:** 2022-11-20

**Authors:** Oussama Jabrane, Driss El Azzab, Pedro Martínez-Pagán, Marcos A. Martínez-Segura, Himi Mahjoub, Mohammed Charroud

**Affiliations:** a Sidi Mohamed Ben Abdellah University, Faculty of Science and Technology, SIGER Laboratory, Fez, BP2202, Morocco; b Department of Mining and Civil Engineering, Universidad Politecnica de Cartagena, Paseo Alfonso XIII, 52, 30203, Cartagena, Spain; c Department of Petrology, Geochemistry and Geological Prospection, Universitat de Barcelona, Martí i Franquès s/n, 08030 Barcelona, Spain

**Keywords:** BERT software, pyGIMLi software, Electrical resistivity imaging data, ERT inversion algorithms, Landslide occurrence, Fez

## Abstract

The electrical resistivity tomography (ERT) technique was conducted for the geophysical survey of a landslide on the southern slope of Jbel Tghat, north of the city of Fez, Morocco. Nine electrical resistivity tomography profiles were implemented to: (a) characterize the geometry of the dipping zone; (b) characterize their internal structures; and (c) highlight the faulting zone between the marly deposits and the conglomerate formation.

The measured data sets were processed using EarthImager™ 2D (Advanced Geosciences, Inc), and BERT (Boundless Electrical Resistivity Tomography) software packages that offer a simple workflow from data import to inversion and visualization, while offering full control over inversion parameters. Moreover, BERT software is a Python-based open-source inversion software package. Both ERT processing software allows obtaining 2D subsurface electrical models associated with the distribution of the subsurface apparent electrical resistivity property, in Ohm.m units. Those 2D subsurface electrical models are retrieved using the same inversion parameters to determine the distribution of geoelectric layers and their defining parameters (e.g., electrical resistivity, thickness, and depth), giving access to certain characteristics exclusive to one of the two processing techniques, comparing the inversion findings to better understand the process's limits, as well as evaluating the capabilities of the two inversion methods.

**Specifications Table**

**Table:** 

Subject	Earth and Planetary Sciences,
Specific subject area	Geophysics, 2D electrical resistivity tomography (ERT), Boundless Electrical Resistivity Tomography (BERT), landslide occurrence.
Type of data	Elevation data (.txt). IRIS Instrument Syscal Pro data (.bin). BERT software data (.UDF). Resistivity data (.dat)
How the data were acquired	In order to collect electrical resistivity tomography data, an IRIS Instrument Syscal Pro resistivity-meter was used in conjunction with a multicore-based connection device capable of concurrently connecting up to 48 electrodes spaced 2 m apart. As a result, the overall length of each ERT profile was 96 m. Electrical Resistivity Tomography (ERT) survey used a dipole-dipole array configuration for electrical resistivity data acquisition.
Data format	Raw Measured Analyzed Processed Filtered Interpreted
Description of data collection	Nine ERT profiles were used to acquire subsurface apparent electrical resistivity values. These ERT profiles, named P1, P2, P3, P4, P5, and P6, have been laid-out to emphasize the interactions among glacis, marls, and conglomerate layers. ERT P1, P2, and P3 profiles are parallel and orientated ENE-WSW. These ERT profiles are separated by half the overall length of the array (48 m), whereas ERT P4, P5, and P6 profiles are perpendicular to P1, P2, and P3 profiles. The entire ERT layout creates a grid, with the objective of allowing correlations in all directions and assessing ERT 2D electrical section consistency. ERT profile P7 is located to the east of P4, P5, and P6. Finally, using a roll-along approach, profiles P8 and P9 were set out in an unoccupied region at a distance of 100 m apart from P7.
Data source location	Institution: Faculty of Sciences and Techniques of Fez, Morocco. City/Town/Region: Tghat-Oued-Fez, Fez, Morocco. Country: Morocco ERT profile First electrode Last electrode P1 34°03′47.6″N; 5°02′08.8″W 34°03′46.6″N; 5°02′12.4″W P2 34°03′48.8″N; 5°02’09.5″W 34°03’47.7″N; 5°02’13.1″W P3 34°03’49.9″N; 5°02′10.1″W 34°03′48.8″N; 5°02′13.6″W P4 34°03′46.9″N; 5°02′11.2″W 34°03′49.8″N; 5°02′12.1″W P5 34°03′46.6″N; 5°02′11.9″W 34°03′49.4″N; 5°02′13.4″W P6 34°03′47.4″N; 5°02′09.1″W 34°03′50.2″N; 5°02′10.7″W P7 34°03′48.7″N; 5°02′02.8″W 34°03′51.6″N; 5°02′04.4″W P8 34°03′50.0″N; 5°01′55.8″W 34°03′52.9″N; 5°01′57.2″W P9 34°03′47.8″N; 5°01′54.4″W 34°03′50.6″N; 5°01′55.9″W
Data accessibility	Repository name: Mendeley Data Data identification number: DOI: 10.17632/38tn7gkgsh.1 Direct URL to data: https://data.mendeley.com/datasets/38tn7gkgsh/1

## Value of the Data

• This ERT dataset helps to determine the distribution of subsurface geoelectric layers, faulting presence, and internal landslide geometry.
• Researchers working on geological mapping in similar areas which are prone to landslide occurrences can utilize these data to identify landslide mechanisms and establish patterns into subsurface electrical resistivity value distribution.
• The ERT data will assist geophysical practitioners in dealing with city expansion plans in areas characterized by potential landslide occurrence risk.
• This dataset may be utilized in a training perspective to gain expertise on the nature of electrical resistivity values associated with natural hazards, specifically landslide mechanisms, allowing for a better understanding of the use of geophysical approaches in the detection and prediction of these hazards.
• These correlations may be used to develop a model considering key elements such as sliding surfaces and displacement factors.

## 1 Objective

This work's dataset focuses on operational and safety challenges linked with technical instability caused by landslide occurrence in urbanized locations. The nine ERT profiles obtained from this data seek to characterize the structure and define the geometry of landslide formations in-depth, as well as the functioning of the role of tectonic activity in the occurrence of such landslides, as well as the joint inversion utilizing two software, which provides an exceptionally flexible solution that works on multiple geometries.

## 2 Data Description

ERT data were stored under .bin file format that provides principally acquired subsurface apparent electrical resistivity values, the injected electrical current (in mA), the measured potential difference (in mV), and the established inter-electrode spacing for each electrical measurement. Where ρ represents the apparent electrical resistivity, in Ohm.m; **Δ**V is the potential difference, in mV; I is the injected current, in mA; AB represents the current electrodes; and MN the potential electrodes; and n is the distance between current and potential electrodes pairs, in m. Elevations along survey profiles were measured and incorporated through a .txt format file to the resistivity data before being converted to a .dat format file. It is worth noting that BERT software also supports the unified data format (.UDF), with topography provided at the end of the file [1].

**Fig. 1:**
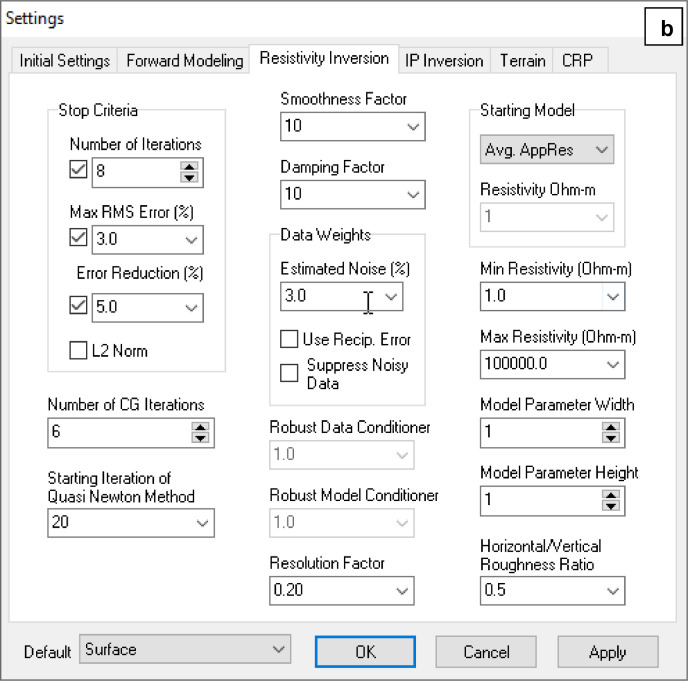
EarthImager screenshots displaying the chosen settings for the inversion process: (a) initial settings, and (b) inversion process settings.

Regarding .bin format files, these data were processed using EarthImager 2D software for inversion processing and retrieving the final subsurface 2D electrical resistivity models under .out format files. For the inversion process was selected smooth fitting mode, with a minimum of eight interactions and a Root Mean Square Error (RMS) value of less than 10% (Fig. 1).

As it has been referred above, BERT software [1] employs a unified data format (.UDF) to organize the information provided by the acquired geoelectrical data. BERT software has the capability to process the acquired geoelectrical data supplied for the most popular and widespread ERT platforms such as AGI SuperSting (.stg), ABEM Terrameter LS (.txt), IRIS Instrument Syscal Pro (.bin), or GeoTom (.flw), as well as other formats such as those generated from Res2Dinv software. The BERT's inversion process is undertaken through finite-element modeling, the whole data processing and visualization uses the pyGIMLi framework [2] in Python using algorithms like the script-based example listed below. The ERTManager module, which is included in pyGIMLi software., was used for the inversion stage. In this way, the final electrical resistivity data files (.dat) with topographic correction were generated by JupyterLab, an extensible environment for interactive and reproducible available in the Anaconda distribution [4], which used Syscal Pro data (.bin), and elevation data (.txt) files. for the added topography data files. Next, is provided the algorithm used for data visualization and inversion through pyGIMLi's ERTManager module:

**import matplotlib.pyplot as plt**

**import numpy as np**

**import pybert as pb**

**import pygimli as pg**

**import pygimli.meshtools as mt**

**from pygimli.physics import** ert

data = pb.load(`TheFileName.bin')

**print**(data)

data[`k'] = ert.createGeometricFactors(data)

k_num = ert.createGeometricFactors(data, numerical=True)

k_ana = ert.createGeometricFactors(data, numerical=False)

data[`k'] = pg.physics.ert.createGeometricFactors(data)

data[`err'] = pg.physics.ert.estimateError(data)

data[`rhoa'] = data[`u'] / data[`i'] * data[`k']

data[`err'] = pg.physics.ert.estimateError(data)

x, z = np.genfromtxt(``TOPOGRAPHYFILE.txt'', encoding=``UTF'',

skip_header=3, unpack=1, usecols=(0, 1), delimiter=``\t'')

**print**(len(x))

**for** i **in** range(data.sensorCount()):

data.setSensor(i, [x[i], 0, z[i]])

data.save(``data.dat'')

**import matplotlib.pyplot as plt**

**import numpy as np**

manager = pg.physics.ert.ERTManager(data, sr=True, verbose =

True)

inversion = manager.invert(lam=**5**, paraMaxCellSize=**1**,

paraBoundary=**0**)

manager.showResult(cMin=**3**, cMax=**200**, xlabel=``x (m)'', ylabel=``z (m)'');

# Source: Authors and pyGIMLi.

## 3 Experimental Design, Materials and Methods

### 3.1 Study site

The research area is located at the crossroads of the pre-Rif mountains and the Sais plain. Tghat-Oued Fez district is a tiny area north of Fez, Morocco, on the southeastern side of Tghat mountain. The study site is dominated by Miocene and Plio-Quaternary deposits with three distinct units [3]. A first colluvial cover unit that forms a fine subsurface layer. A second marly unit containing concrete gypsum, and a final conglomerate formation composed of detrital terrigenous and siliciclastic elements.

There is a gradual landslide (up to a few centimeters per year), which can be detected by minor scarps, transverse fractures and ridges, and other surface indicators. It mobilizes a vast mass all along the slope of Tghat-Oued Fez district, which has a varying slope depending on different locations and is quite average (10-25°).

**Fig. 2:**
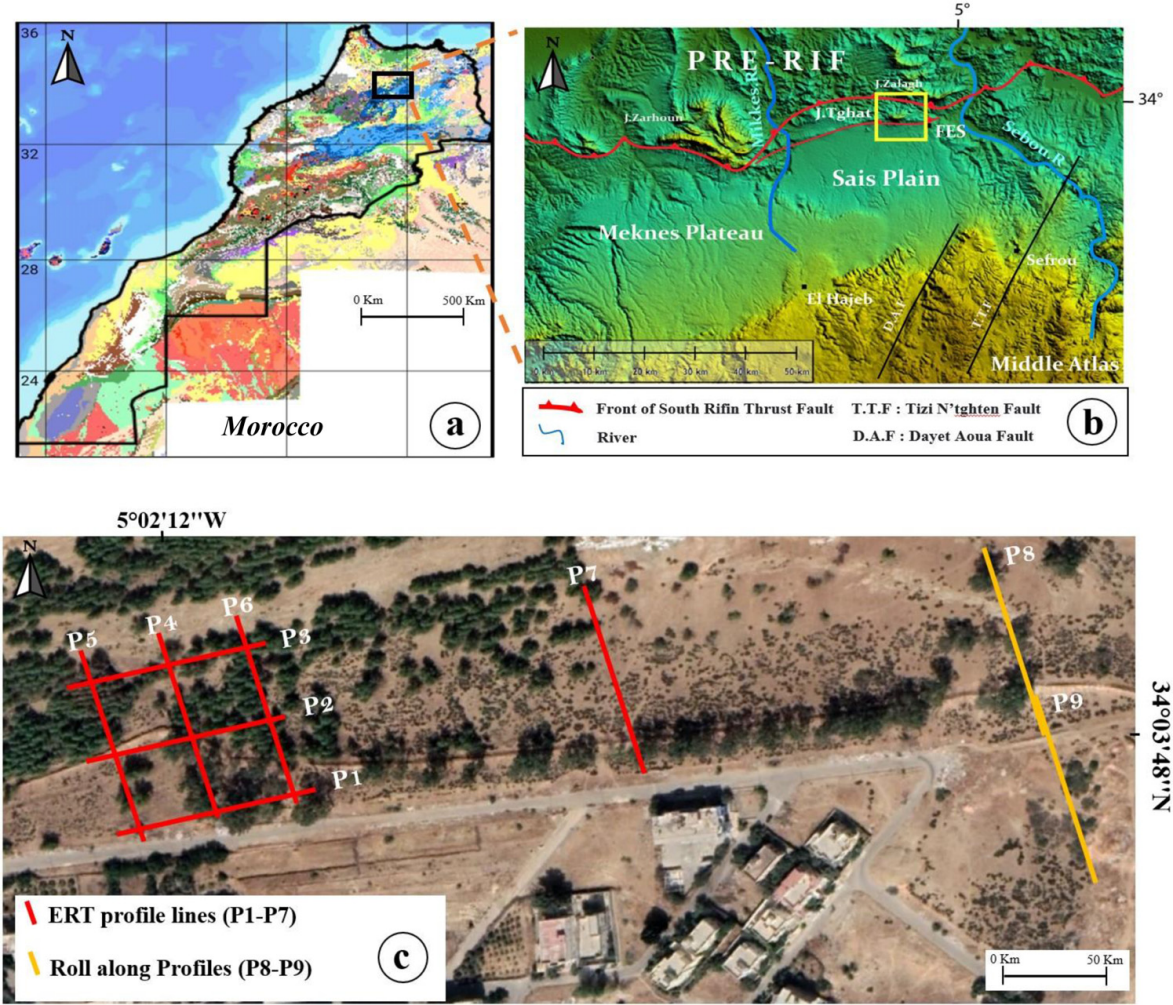
(a) Map of Morocco showing the study area. (b) Faulting system affecting the Tghat-Oued Fez district. (c) ERT profile layout at the study area.

Fig. 2 shows the ERT profiles layout which were conducted to define the local subsurface geology and assist in the understanding of the internal sliding mechanism taking place at that site. Thus, ERT profiles provide 2D electrical sections, whose final electrical resistivity values are associated with subsurface materials, fractures, interfaces, faults, etc., at the final interpretation/visualization stage.

As it was stated above, the generated 2D electrical sections were obtained with RMS values below 10%, which guarantee the appropriate quality of the final electrical resistivity data generated by means of the inversion process. In fact, the final number of iterations for each inversion process is decided by the RMS error, which stops the iteration process once the RMS value reaches a previously stablished value (e.g., 10%).

**Fig. 3:**
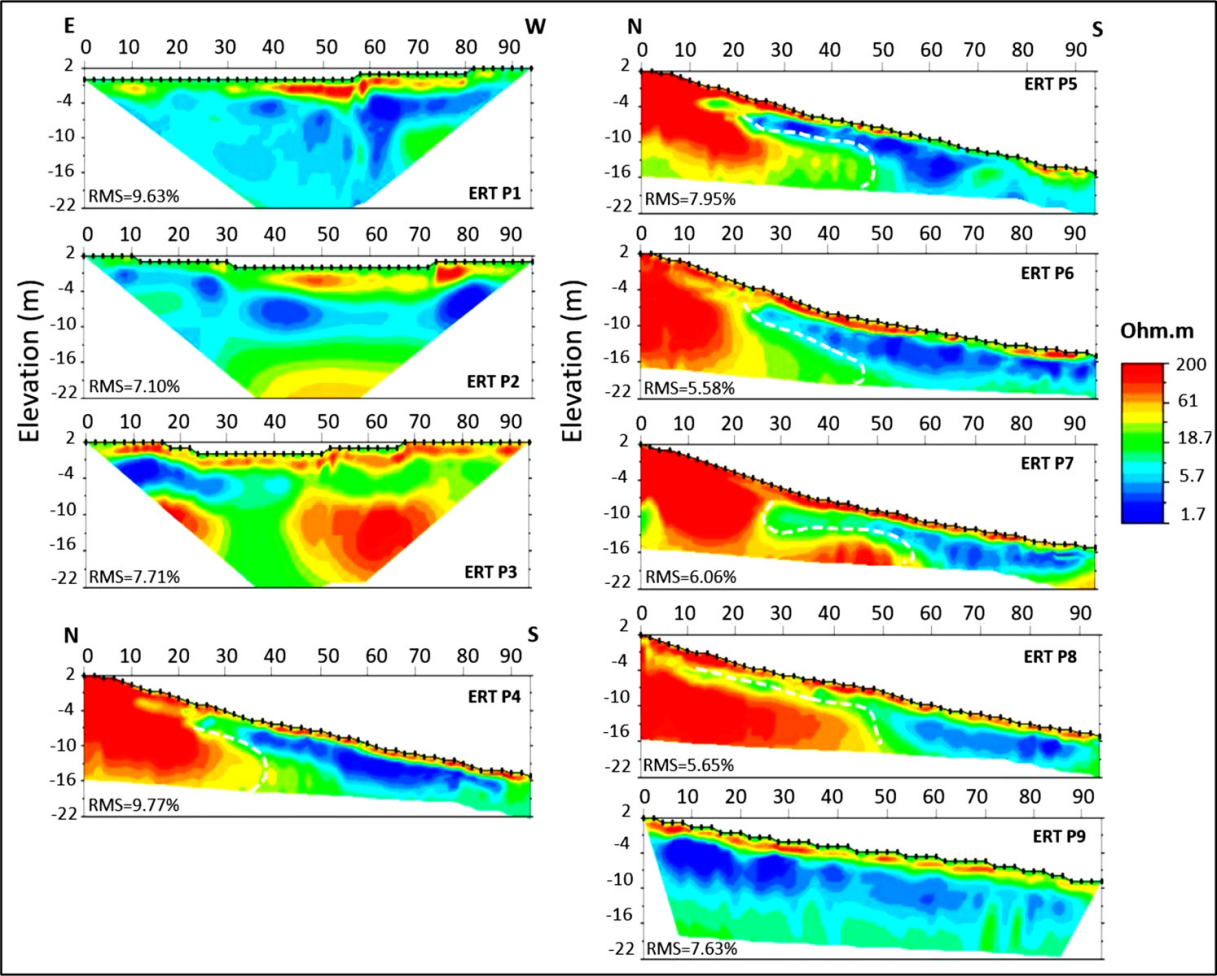
Inverted ERT 2D electrical sections generated by EarthImager software.

As a result, the shown 2D electrical section from ERT P1 profile (Fig. 3) depicts two distinguishable subsurface zones characterized by their associated ranges of electrical resistivity values. ERT P2 and P3 2D electrical sections (Fig. 3) emphasize the presence of three main resistive zones. A first surficial and resistive layer associated with the colluvial cover unit, marked by high resistivity values (100 to 200 Ohm-m). Then, there is a second and more conductive layer (between 2 to 20 Ohm-m) associated with the marly formation, which overlies a third and very resistive (up to 200 Ohm-m) layer associated with the conglomeratic formation, both of which are evident at 10 m deep.

The ERT P4, P5, and P6 2D electrical sections (Fig. 3) have very identical formation layouts as well as horizontal and vertical distribution. In the beginning, there is a subsurface resistive layer (100 to 200 Ohm-m). that represents glacis deposits. In contrast, a conductive layer with low electrical resistivity values (2 to 10 Ohm-m). occurs at 4 m depth. This conductive layer determines the presence of the green marl formation. Finally, hardened conglomerates deposits are the third formation with a highly refractory layer. The thickness of this more resistive layer (200 Ohm-m) fluctuates throughout the electrical section based on its geometry, exposing a dipping-shaped structure oriented towards the south. The passage of a fault marks the zone of passage between the highly resistive conglomerates and the conductive marls; this zone corresponds to the sliding surface.

The ERT P7 and P8 2D electrical sections (Fig. 3) were carried out further east from the ERT P4, P5, and P6 profile set up to determine lateral changes and possible continuation of the landslide and surface contact with the marly formation. These 2D electrical sections, as illustrated in Fig. 3, exhibit the same pattern in terms of electrical layers as it has been discussed above. They enable the identification of resistant surfaces, the continuity of the marly formation, and the direction of the landslide surface towards the south. Additionally, it is noticeable in those 2D electrical sections of the P7 and P8 profiles (Fig. 3) the presence of conductive areas penetrating to the northern part of the area affected by the landslide, something that might explain the presence of water content. These conductive zones point out the active area of the landslide, emphasizing the influence of subsurface water on the mechanisms of slope instability.

In contrast, the ERT P9 2D electrical section (Fig. 3) displays a horizontally stratified structure of the principal three electrical layers. The thickness of the most surficial resistive layer at this point, associated with glacis cover deposits, is thicker than it is showed in the previous ERT profiles. Secondly, a significantly larger conductive marly layer with a depth of over 18 m and a consistent geometry along the entire profile. Furthermore, at a depth of 20 m, begins the appearance of a more resistant level, which is associated to the occurrence of conglomerate formation. The fact that this last profile is located further south than the others explains the depth, revealing the landslides southern boundary.

## 4 BERT Profiles

**Fig. 4:**
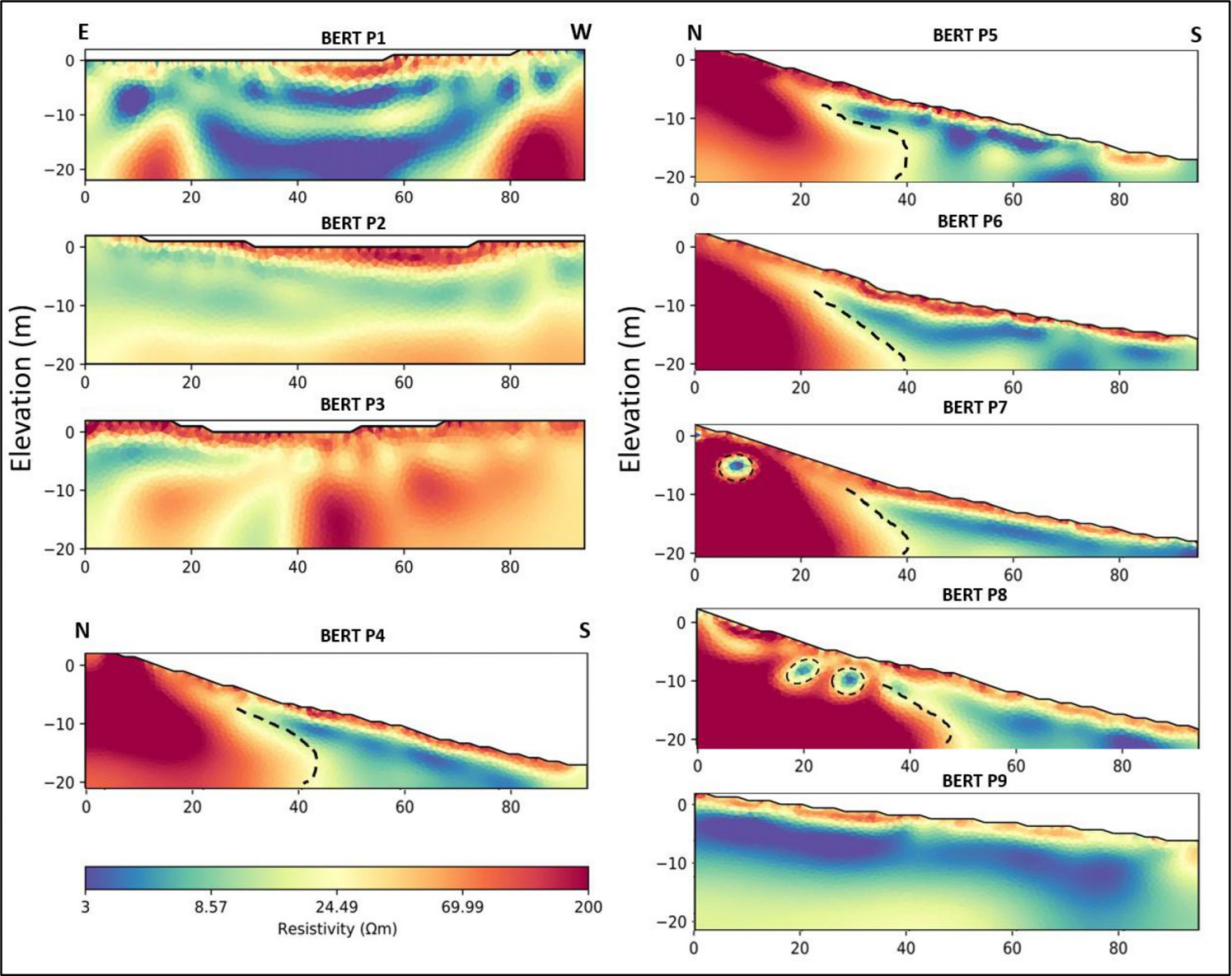
Inverted ERT 2D electrical sections generated by BERT software.

The Python-based pyGIMLi software has been used for BERT inversion processing and visualization stages [2]. The generated ERT 2D electrical sections exhibit a chi-squared misfit close to one. To enhance data fit, the regularization parameter (LAMBDA) for all profiles was set at a value of 5, resulting in a relative RMS error of about 3% for all profiles (Table 1). These 2D electrical sections (Fig. 4) exhibit a good correlation to those obtained by EarthImager 2D with reference to subsurface electrical resistivity distribution, thus enabling to reach the same interpretation concerning internal formations and landslide mechanisms.

**Table 1:** Final BERT inversion processing values for each retrieved ERT 2D electrical section (number of iterations, chi-square, and RMS error).

	Inversion iteration	chi² (initial and final)	RMS
BERT P1	9	113.19/4.46	3.02%
BERT P2	4	12.91/0.6	3%
BERT P3	6	4.41/0.64	3.01%
BERT P4	5	21.47/1.17	3.02%
BERT P5	6	50.77/13.35	3.03%
BERT P6	5	37.73/0.35	3.2%
BERT P7	20	134.19/3.99	3.02%
BERT P8	10	25.94/0.99	3.02%
BERT P9	5	37.46/0.56	3.01%

The assessment of the nine ERT profiles processed by the BERT program reveals that there is a general similarity between these, and the resultant ERT profiles processed by EarthImager using the same standard inversion parameters. On the first three east-west ERT profiles, the number of electrical layers emphasized is the same, as is the internal arrangement of formations: a resistive glacis cover, then a homogenous and more conductive marly layer, and finally, at a depth of 20 meters, the development of harder and more resistive conglomerate deposits.

The lithological distribution and formation thickness of the BERT P4, P5, and P6 2D electrical sections (Fig. 4) are remarkably similar to those obtained by EarthImager. Consequently, it is noticeable the existence of a more resistive layer from 2 to 4 m depth along the ERT profiles that represent glacis deposits. Then, a conductive layer underlies in the center of the profiles heading south, which is associated with marl formation deposits. Besides, at the bottom of the 2D electrical sections, a third and more resistive layer, associated with more compact conglomerate deposits, is identified. The thickness of this more resistive layer varies across the electrical section showing a thicker area to the north, in where a dipping structure is revealed to the south. Furthermore, the presence of a fault structure defines a transitional zone between the highly resistive conglomerates and the more conductive marls, corresponding to this transitional zone to the sliding surface.

The BERT P7 and P8 2D electrical sections (Fig. 4) exhibit the same style as that obtained by EarthImager for the same 2D electrical sections (Fig. 3). Therefore, it could be observed the same tendency of sliding towards the south. Moreover, it is noticeable, again, the presence of tiny and round-shape conductive zones on the upper left side of ERT P7 and P8 electrical sections, particularly on the ERT P8 electrical section, within the zone affected by the landslide between the surface formation and the conglomerates, which might reflect the presence of internal water content. These conductive zones might be associated with the most active part of the mechanism, indicating that the presence of water has a significant influence on the landslide mechanism triggering and generating slope instability.

These reusable ERT data are available in the Mendeley repository, in which someone could access a ".rar" folder consisting of ERT datasets included in the original .bin and .dat formats. These datasets are properly labeled with the assigned ERT profile number and name. Moreover, the topography is included by means of a .txt file for BERT inversion.

## Ethics Statements

Authors state that the data supporting their paper meet the data requirements of the journal, ensuring that they have written an original work, not submitting this paper for consideration in another journal. The authors, also, declare no conflict of interest, or any financial of this work. Moreover, the authors declare no potential ethical issues in the paper or during its writing.

## CRediT Author Statement

**Oussama Jabrane, Driss El Azzab, Himi Mahjoub** and **Mohammed Charroud:** Conceptualization, Methodology, Software; **Pedro Martínez-Pagán** and **Marcos Martínez-Segura:** Inversion Software and Validation; **Oussama Jabrane, Driss El Azzab** and **Mohammed Charroud:** Investigation and Resources; **Oussama Jabrane:** Data Curation; **Oussama Jabrane:** Writing – original Draft Preparation; **Driss El Azzab** and **Pedro Martínez-Pagán:** Writing – review & editing; **Driss El Azzab** and **Pedro Martínez-Pagán:** Supervision and Project Management.

## Declaration of Competing Interest

The authors declare that they have no known competing financial interests or personal relationships that could have appeared to influence the work reported in this paper.

## Data Availability

Electrical Resistivity Tomography datasets for landslide detection. A case study in Tghat-Fez (Morocco) (Original data) (Mendeley Data).

## References

[1] Günther, T.; Rücker, C. Boundless Electrical Resistivity Tomography BERT2—The User Tutorial. 2019. Available online: http://www.resistivity.net/download/bert-tutorial.pdf. Accessed August 1st, 2022.

[2] Rücker C., Günther T., Wagner F.M. (2017). pyGIMLi: An open-source library for modelling and inversion in geophysics. Comput. Geosci..

[3] Charroud M, Cherai B, Benabdelhadi M, Charroud A, El Moutaouakkil N, Falguères C, Lahrach A (2006). Sedimentary evolution of a fore-chain Sais basin during plio-quatrenary and modalities of tectonic inversion (Sais basin, Morocco). Geophys. Res. Abst. Eur. Geosci. Union.

[4] Anaconda Documentation. Anaconda individual edition. [online]. 2022. [date of reference February 21st of 2022]. Available online: https://docs.anaconda.com/anaconda. Accessed October 23th, 2022.

